# Development of a protein–ligand-binding site prediction method based on interaction energy and sequence conservation

**DOI:** 10.1007/s10969-016-9204-2

**Published:** 2016-07-11

**Authors:** Hiroto Tsujikawa, Kenta Sato, Cao Wei, Gul Saad, Kazuya Sumikoshi, Shugo Nakamura, Tohru Terada, Kentaro Shimizu

**Affiliations:** Department of Biotechnology, The University of Tokyo, 1-1-1 Yayoi, Bunkyo-Ku, Tokyo 113-8657 Japan

**Keywords:** Protein structure, Protein–ligand binding, Binding site prediction, Interaction energy, Sequence conservation

## Abstract

We present a new method for predicting protein–ligand-binding sites based on protein three-dimensional structure and amino acid conservation. This method involves calculation of the van der Waals interaction energy between a protein and many probes placed on the protein surface and subsequent clustering of the probes with low interaction energies to identify the most energetically favorable locus. In addition, it uses amino acid conservation among homologous proteins. Ligand-binding sites were predicted by combining the interaction energy and the amino acid conservation score. The performance of our prediction method was evaluated using a non-redundant dataset of 348 ligand-bound and ligand-unbound protein structure pairs, constructed by filtering entries in a ligand-binding site structure database, LigASite. Ligand-bound structure prediction (bound prediction) indicated that 74.0 % of predicted ligand-binding sites overlapped with real ligand-binding sites by over 25 % of their volume. Ligand-unbound structure prediction (unbound prediction) indicated that 73.9 % of predicted ligand-binding residues overlapped with real ligand-binding residues. The amino acid conservation score improved the average prediction accuracy by 17.0 and 17.6 points for the bound and unbound predictions, respectively. These results demonstrate the effectiveness of the combined use of the interaction energy and amino acid conservation in the ligand-binding site prediction.

**Electronic supplementary material:**

The online version of this article (doi:10.1007/s10969-016-9204-2) contains supplementary material, which is available to authorized users.

## Introduction

It is well known that the biological function of many proteins depends on binding to small molecules, termed ligands. Therefore, the function of a protein can be inferred by determining what kinds of ligands it binds. In addition, in recent years, the three-dimensional (3D) structures of proteins have been used in structure-based drug design. Because ligands bind to specific sites on the surfaces of proteins, identification of the ligand-binding sites is an essential step in these studies. Various methods have been developed for ligand-binding site prediction, and because ligand-binding sites are often located in large depressions (pockets) on protein surfaces, many of these prediction methods use 3D protein structures to predict ligand-binding sites. These structure-based methods can be largely classified into two groups: (a) purely geometric methods [1–4] and (b) energetic methods [5, 6].

In the purely geometric approaches, the ligand-binding site is presumed to be located within the largest pocket on the protein surface. However, when the size of the pocket is larger than that of the ligand, the exact binding site cannot be easily predicted. Furthermore, the spatial range of the detected pocket varies between the prediction methods, reflecting differences in the definition of the pocket among the methods.

The energetic methods are based on the concept that a ligand binds the site where the interaction energy with the protein is minimal. To search for such a site, ligands are virtually placed on the protein surface, and the interaction energy with protein atoms is calculated at each position to estimate the stability of the binding site.

Laurie and Jackson’s Q-SiteFinder [6] is one of the most successful energetic methods for predicting ligand-binding sites. Q-SiteFinder first places methyl probes (−CH_3_) in a grid around a protein molecule and calculates van der Waals interaction energy between the atoms of the protein and probes. Probes with low energy are then clustered. The clustering is repeated until a cluster with the total interaction energy of the probes being lower than a defined threshold is obtained. Clusters thus obtained are ranked according to the total energy, and the cluster with the lowest total energy is expected to be the most appropriate for the ligand-binding site.

However, energy-based methods are not always superior to purely geometric methods, and adequate precision cannot be achieved by only defining the “energetically stable site.” Therefore, improvement of the precision of the prediction has been attempted by combining new information that indicates ligand-binding site-like features with the conventional methods.

Two sets of information have often been used in practice. One is amino acid frequency around ligand-binding sites. Amino acids on a protein surface are more likely to be in the ligand-binding sites than those buried in the protein. Accordingly, the likelihood of a site being a ligand-binding site can be quantified by evaluating the frequencies of the 20 amino acids for the site and comparing them with those for the protein surface and protein interior [7]. The other set of information is amino acid conservation. Because ligand-binding sites are the most important sites for expressing protein function, there is a strong tendency for amino acids around binding sites to be conserved among homologues [8].

Several approaches have been attempted to improve the precision of prediction by applying one of these two sets of information to conventional methods. Kulharia et al. [7] reported improvement in prediction precision by the inclusion of amino acid frequency in the prediction by Q-SiteFinder. In contrast, sequence conservation (amino acid conservation) has been employed in purely structure-based methods. In LIGSITEcsc [4], multiple pockets obtained from a grid search are re-ranked based on the degree of amino acid conservation in the proteins. Concavity [9] is a method that employs amino acid conservation in pocket searching; this method differs from LIGSITEcsc in incorporating conservation information directly into the search for pockets rather than using conservation information to postprocess predicted pockets. Capra et al. [9] states that concavity outperforms LIGSITEcsc because of this difference.

In the present study, we developed a prediction method that combined amino acid conservation with an energy-based method. The energy-based pocket search is similar to Q-SiteFinder and our previous work [18], and the amino acid conservation is directly incorporated into the ranking of ligand-binding sites, as in concavity. As for the energy calculation, we use van der Waals energy as an interaction energy between a carbon atom probe and the protein. This energy is not related to a physical protein–ligand-binding energy and is used only as a tool to identify and rank protein cavities. Such an abstraction can be useful for coping with various types of ligands, particularly when the ligands are not known in advance. We collected a wide range of test data and performed a general assessment of prediction precision. Our method successfully predicted binding sites with a higher precision than a conventional energy-based method, Q-SiteFinder, clarifying the effect of amino acid conservation on the prediction precision.

## Materials and methods

### Dataset construction

Two sets of protein structures corresponding to each other are developed in the present study: ligand-bound structures in which ligands are bound to proteins and ligand-unbound structures that are ligand-free structures of proteins in the ligand-bound structure set. The list of each protein set was obtained from LigASite [10], a protein–ligand-binding site database. A non-redundant version of the list including 391 protein pairs was used. Among them, two proteins were omitted because ligand-bound coordinates could not be found. Also, as we only consider homo-multimer proteins, seven hetero-multimer proteins were omitted. Hence, in the present study, 382 protein pairs were used in total. For some pairs, there are multiple ligand-bound structures in the list. In those cases, one structure was randomly selected from the list for each pair. Biological oligomeric assemblies were obtained from the predicted quaternary structures using Proteins, Interfaces, Structures and Assemblies (PISA) [11]. The protein structures used in this study were determined by X-ray crystallography with a resolution of ≤2.4 Å and *R*-factor ≤0.25.

According to LigASite [10], the dataset is limited to binding sites for the clusters of non-water molecules appearing in the HETATM records of Protein Data Bank (PDB) entries within 4 Å that consist of at least 10 heavy atoms and that make at least 70 interatomic contacts with protein atoms. This method was adopted to select biologically relevant ligands in this study. As for the quality of ligands and ligand-binding site structures of PDB, we performed the analysis of B-factor and Local Ligand Density Fit (LLDF). The results of these analyses and a list of PDBIDs of 382 ligand-bound and ligand-unbound structures used in the present study are shown in Online Supplementary Material.

### Prediction method outline

**Fig. 1:**
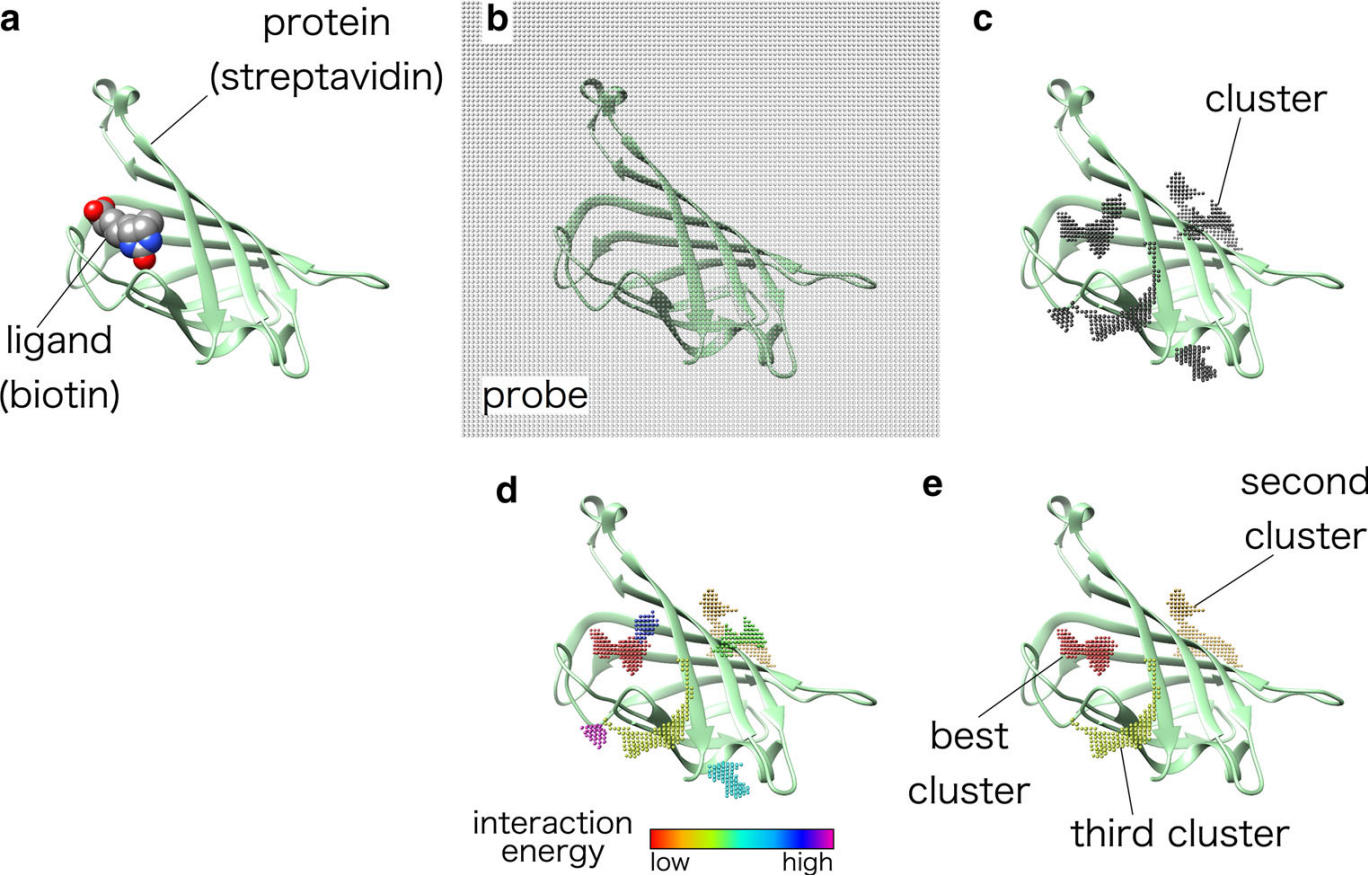
Ligand-binding site prediction. **a** An example using the protein (streptavidin) and the ligand (biotin), PDBID: 1stp. **b** Placement of probes around the protein and calculation of interaction energies between probes and the protein. **c** Forming clusters of probes. **d** Ranking clusters. **e** Clusters of the top three ranking. Steps related to amino acid conservation are not shown

Our prediction method includes the following steps, illustrated for the example of biotin binding of streptavidin in Fig. 1a.

1. Addition of hydrogen atoms and construction of missing side chains of proteins.
2. Placement of carbon atom probes around the protein (Fig. 1b).
3. Calculation of van der Waals energy.
4. After calculation of interaction energy of all the probes, if a probe with lower interaction energy is found or a probe with similarly low interaction energy is found within 1 Å of another probe, cluster them together. Expand the size of a cluster until it reaches a defined size (Fig. 1c).
5. Calculation of amino acid conservation.
6. Weighting of interaction energy with amino acid conservation.
7. Ranking of clusters in ascending order according to total probe interaction energy within a cluster (Fig. 1d, e).

#### Addition of hydrogen atoms and construction of missing side chains of proteins

Normally, 3D coordinates obtained from PDB and PISA does not include hydrogen atoms if they were determined by X-ray crystallography. In the proposed method, a hydrogen addition tool, *protonate*, in the molecular dynamics software AMBER10 was used to add hydrogen atoms to proteins for calculating van der Waals energy between all atoms and probes of a protein. With regard to proteins with side chains whose complete atomic coordinates were not registered, a side chain modeling tool, SCWRL3 [12], was used to reconstruct the missing side chain before the addition of hydrogen atoms.

#### Placement of carbon atom probes around the protein

Carbon atom probes were placed in a grid around a protein with 0.5 Å intervals. The van der Waals interactions commonly appear in the protein–ligand binding, and we calculated the van der Waals interaction energy between a carbon atom and the protein. This interaction energy is not directly related to real ligand-binding energy; it is only used for obtaining a position with minimum energies. Carbon atoms are simple and have lower precision than all-atom methane or methyl probes; however, in this model, coordinates of hydrogen atoms that change because of atomic rotation need not be considered, indicating an advantage of reduced computational complexity.

#### Calculation of van der Waals energy

Van der Waals energy between probe–protein atoms was calculated using the equation, Lennard–Jones 6–12 potential *E*^van^:

$$E_{i,j}^{van} = \sqrt {\varepsilon_{j} \varepsilon_{j} } \times \left\{ {\left( {\frac{{R_{i} + R_{j} }}{{r_{ij} }}} \right)^{12} - 2 \times \left( {\frac{{R_{i} + R_{j} }}{{r_{ij} }}} \right)^{6} } \right\}$$

In this equation, *i* and *j* represent probe and protein atom, respectively, whereas *r*_ij_ indicates the distance between two atoms, *R* the van der Waals distance (Å), and *ε* the van der Waals depth (kcal/mol). Each atom has its unique *R* and *ε*. In the present study, van der Waals energy was calculated based on a force field parameter, parm94 [13]. To reduce computation cost, van der Waals energy between a probe and protein atoms located within 10 Å of the probe was calculated for each probe.

#### Clustering of probes

Interaction energy with protein atoms was calculated for each probe, and probes were clustered using the value of the interaction energy. First, the probe with the lowest energy was found, and its energy was used as the energy threshold. Next, the energy threshold was increased by 0.1 kcal/mol, and probes with energy lower than the energy threshold were searched for. If such probes were found, probes with energy lower than the energy threshold and located within 1 Å of that probe were clustered together. The cluster can be merged during this step. The distance between clusters was defined as the distance between two probes in the clusters nearest to each other. When no more probes were found for clustering, the energy threshold of the search was broadened by 0.1 kcal/mol. The maximum number of probes that could be included in a cluster was defined as *P*_num_, and when the total number of probes within a cluster reached *P*_num_, the clustering process was completed.

#### Calculation of amino acid conservation

PSI-BLAST, with up to three iterations against the NCBI non-redundant (nr) database, was used to compare the sequence similarity between the amino acid sequence of the target protein and the sequences of its homologues, and the resulting position-specific scoring matrix was used to calculate amino acid conservation.

When *P* indicates amino acid frequency in a protein and *Q* indicates the amino acid frequency in the background, the Kullback–Leibler divergence can be obtained [14] as the difference between the mean frequency and both *P* and *Q*. Furthermore, the Jensen–Shannon divergence may be derived [15] by calculating the mean of Kullback–Leiber divergences and was defined as the degree of amino acid conservation, *C*_score_.

*C*_score_ and *E*^van^ [in Eq. (1)] obtained as described above were used for calculating the weighted score. The weighted score of probe *i* is defined as follows:

1 $$E_{i} = \sum\limits_{j = 1}^{n} {\left( {w_{1} E_{i,j}^{van} + w_{2} Cscore_{j} + w_{3} E_{i,j}^{van} Cscore_{j} } \right)}$$

where *n* is the number of the protein atoms that interact with probe *i* and *j* denotes their index. *C*_score_ is defined for each amino acid residue and is applied to all the atoms of that residue.

In this equation, *w*_1_, *w*_2_, and *w*_3_ indicate the weights on the respective terms. We changed the values of *w*_2_ and *w*_3_ with the value of *w*_1_ fixed to 0 or 1. Only *E*^van^ of protein atoms within 10 Å of a probe and *C*_score_ were weighted. This is because the amino acids distant from a ligand were considered to have low influence on the stable binding of the ligand. Accordingly, for the second term of Eq. (1), amino acids found ≤6 Å from a probe was used by calculating *C*_score_, and amino acids located >6 Å away from the ligand were not used.

#### Ranking of clusters

All clusters obtained were arranged in the ascending order of the sum of the probe scores *E*_*i*_ in them. For multimeric proteins, similar clusters with similar energy values were obtained for corresponding sites of all chains. In these cases, we consider only one cluster with the lowest energy value among them.

### Methods for assessment and comparison of prediction results

Two measures were used to assess the performance: the proportion of predicted clusters that spatially overlap with actual ligands above chosen threshold (hereafter referred to as ligand-binding space prediction) and the degree of agreement between amino acids around a cluster and the exact amino acid around the ligand (hereafter referred to as ligand-binding residue prediction).

#### Assessment of the results of ligand-binding space prediction

For ligand-bound structures, a method of calculating the proportion of clusters that spatially cover ligands [6] was used for performance assessment. We determine that a probe in a cluster overlaps with the ligand if the probe is located within 1.6 Å from one of the heavy atoms of the ligand. We adopted the same value 1.6 Å as that of Q-SiteFinder to compare the performance directly.

The term “precision” used here defines the proportion of probes in a cluster that overlap with ligands. When the value is ≥25 %, the cluster is regarded as a success. A precision of 100 % means that all probes in the cluster are located within 1.6 Å of one of the heavy atoms of the ligand, and when the cluster is larger, the precision decreases. In some cases, the prediction results even with small precision values are useful; however, if we regard a cluster with a precision >0 % as a success, a very large cluster that accidentally includes a small number of probes within 1.6 Å from the ligand will also be a success. To avoid such cases, we referred to Laurie et al. and defined a precision threshold of ≥25 %.

As for the ligand-unbound structures, the ligand coordinates are not available, so ligand coordinates should be copied to the ligand-unbound structure from its pair (i.e., ligand-bound structure) to obtain a pseudo-binding site. However, superposition of proteins is difficult when large structural changes occur upon ligand binding. Therefore, we instead used a method that compares residues around a ligand-binding site that is generally applicable to ligand-unbound structures.

#### Assessment of the results of ligand-binding residue prediction

The assessment method of the results of ligand-binding residue prediction employed in the present study, which is similar to the assessment score calculation used in the ligand-binding site prediction category [16] of the Critical Assessment of Structure Prediction (CASP, http://www.predictioncenter.org/), is as follows:

1. Residues located within 5 Å of each probe in a cluster are regarded as the ligand-binding residues of the cluster.
2. Calculate the following values: the number of residues predicted correctly as ligand-binding residues (true positive), the number of residues correctly predicted as nonligand-binding residues (true negative), the number of residues incorrectly predicted as ligand-binding residues (false positive), and the number of residues incorrectly predicted as nonligand-binding residues (false negative). Finally, calculate scores, *S*_residue_, defined as the proportion of correctly predicted ligand-binding residues among all the positively predicted ligand-binding residues and Matthew’s correlation coefficient (MCC) based on the above values.

Information about residues around ligands (i.e., correct ligand-binding residues) was obtained from LigASite. In addition to MCC, we used *S*_residue_ as an assessment score to indicate the proportion of correctly predicted residues around ligands.

#### Parameter selection using cross-validation

In ligand-binding space prediction, fivefold cross-validation was performed using the 382 ligand-bound structures; these structures were randomly divided into five subsets of equal size, and four of them were used for training parameters as described in the Results section and then one was used for testing. This process is repeated five times, each time using a different subset for testing and the other four subsets for training. The performance was assessed as the average of each success rate and average precision. Ligand-binding residue prediction can be used to assess the results of prediction of both ligand-bound and ligand-unbound structures. Therefore, fivefold cross-validation was first performed using ligand-bound structures, and the parameters showing the best performance were further used to predict residues for ligand-unbound structures. The procedure was evaluated with respect to the successful prediction of correct ligand-binding sites using ligand-unbound proteins.

## Results

The parameters for the proposed method were the maximum number of probes within a cluster (*P*_num_) and the weights for the amino acid conservation (*w*_1_, *w*_2_, *w*_3_) in the probe energy [Eq. (1)].

In ligand-binding space prediction, the optimum value *P*_num_ was searched first at intervals of 100 in the range 100–600, and fivefold cross-validation was performed for the 382 ligand-bound structures. *P*_num_ = 500 was selected because it yielded the highest success rate.

Next, with *P*_num_ = 500, the weights (*w*_1_, *w*_2_, *w*_3_) were determined by fivefold cross-validation, and the mean values of the highest success rate and the corresponding mean precision in each cross-validation were calculated. Among the five parameter sets with a highest success rate at each test step in the cross-validation, the most frequently observed combination (*w*_1_, *w*_2_, *w*_3_) = (1, −0.05, 9) was used as the optimal weights.

Table 1 shows the results of ligand-binding space prediction. Excluding proteins containing over 10,000 atoms, which are not accepted by the Q-SiteFinder server, 342 ligand-binding sites were used for the assessment as their ligand-bound and ligand-unbound structures were both available. The predicted clusters are ranked according to the score defined in Eq. (1). The method denoted as “Our method (without *C*_score_)” uses the score with *w*_2_ and *w*_3_ zero. In Table 1, “Top1” indicates the best cluster per structure and “Top3” indicates a cluster in the three best clusters per structure.

**Table 1:** Comparison of prediction results from our proposed method and Q-SiteFinder (for ligand-binding space prediction, 342 ligand-bound structures)

	Top1, precision ≥25 %		Top1, precision >0 %		Top3, precision ≥25 %	
	Ratio (%)	Average precision (%)	Ratio (%)	Average precision (%)	Ratio (%)	Average precision (%)
Our method	**74.0**	64.4	**80.1**	**60.5**	**88.0**	66.4
Our method (without *C*_score_)	57.0	59.7	66.1	53.5	73.7	62.7
Q-SiteFinder	52.0	**66.0**	58.5	60.2	71.9	**66.9**

The results of “our method” are highlightened in bold

“Precision” indicates the proportion overlapping with ligands, and the overlap indicates that a probe in a cluster is located within 1.6 Å from one of the ligand heavy atoms. The predicted clusters are ranked according to the score defined in Eq. (1). The condition “Top1, precision ≥25 %” indicates the best cluster with precision ≥25 %, “Top1, precision >0 %” indicates the best cluster with precision >0 %, and “Top3, precision ≥25 %” indicates that at least one cluster in the best three clusters has precision ≥25 %. “Ratio” is the number of proteins with the specified condition divided by the number of proteins in the dataset. “Average precision” is the average precision of the proteins with the specified condition

With regard to the ligand-binding space prediction, the success rate was confirmed to be higher than that of Q-SiteFinder under each criterion of success. More specifically, the success rate for the clusters in the first prediction rank with precision ≥25 % increased from 52.0 to 74.0 % and that for the clusters within the first three prediction ranks with precision ≥25 % increased from 71.9 to 88.0 %. With regard to average precision, sometimes Q-SiteFinder was superior depending on the criteria of success; however, even in those cases, the values from the two methods were similar. When our method with amino acid conservation [*C*_score_ in Eq. (1)] was compared with that without conservation, the success rate for the cluster in the first prediction rank with precision ≥25 % increased from 57.0 to 74.0 % and that for the clusters within the first three prediction ranks with precision ≥25 % increased from 73.7 to 88.0 %. These results show that the effect of using amino acid conservation was pronounced.

Table 2 shows the results of ligand-binding residue prediction. Here 348 ligand-unbound structures were used for the assessment as their ligand-bound and ligand-unbound structures were both available. As in ligand-binding space prediction, the predicted clusters are ranked according to the score defined in Eq. (1). The method denoted as “Our method (without *C*_score_)” uses the score with *w*_2_ and *w*_3_ zero. With regard to ligand-binding residue prediction, success rate, average precision, and average MCC were confirmed to be higher than those of Q-SiteFinder under all the criteria of success.

**Table 2:** Comparison of prediction results from our proposed method and Q-SiteFinder (for ligand-binding residue prediction, 348 ligand-unbound structures)

	Top1, *S*_residue_ ≥25 %		Top1, *S*_residue_ ≥0 %		Top3, *S*_residue_ ≥25 %		Top1
	Ratio (%)	Average *S*_residue_ (%)	Ratio (%)	Average *S*_residue_ (%)	Ratio (%)	Average *S*_residue_ (%)	Average MCC
Our method	**73.9**	**61.3**	**86.2**	**54.3**	**85.6**	**61.6**	**0.51**
Our method (without *C*_score_)	56.3	58.2	74.7	47.0	76.1	58.6	0.39
Q-SiteFinder	53.4	60.4	69.3	49.2	74.4	60.1	0.33

The results of “our method” are highlightened in bold

For ligand-binding residue prediction, *S*_residue_ is used as the measure of precision. The condition “Top1, *S*_residue_ ≥25 %” indicates the best cluster with *S*_residue_ ≥25 %, “Top1, *S*_residue_ >0 %” indicates the best cluster with *S*_residue_ >0 %, and “Top3, *S*_residue_ ≥25 %” indicates that at least one cluster in the best three clusters has the *S*_residue_ ≥25 %. “Ratio” is the number of proteins with the specified condition divided by the number of proteins in the dataset. “Average precision” is the average precision of the proteins with the specified condition

More specifically, the success rate for the cluster in the first prediction rank with *S*_residue_ ≥ 25 % increased from 56.3 to 73.9 % and that for the clusters within the first three prediction ranks with *S*_residue_ ≥ 25 % increased from 74.4 to 85.6 %. Average *S*_residue_ was found to be greater than Q-SiteFinder for all criteria. When our methods with amino acid conservation were compared with that without conservation, the success rate for the cluster in the first prediction rank with *S*_residue_ ≥ 25 % was improved from 53.4 to 73.9 % and that for the clusters within the first three prediction ranks with *S*_residue_ ≥ 25 % increased from 76.1 to 85.6 %. These results show that, again, the effect of using amino acid conservation was pronounced.

**Fig. 2:**
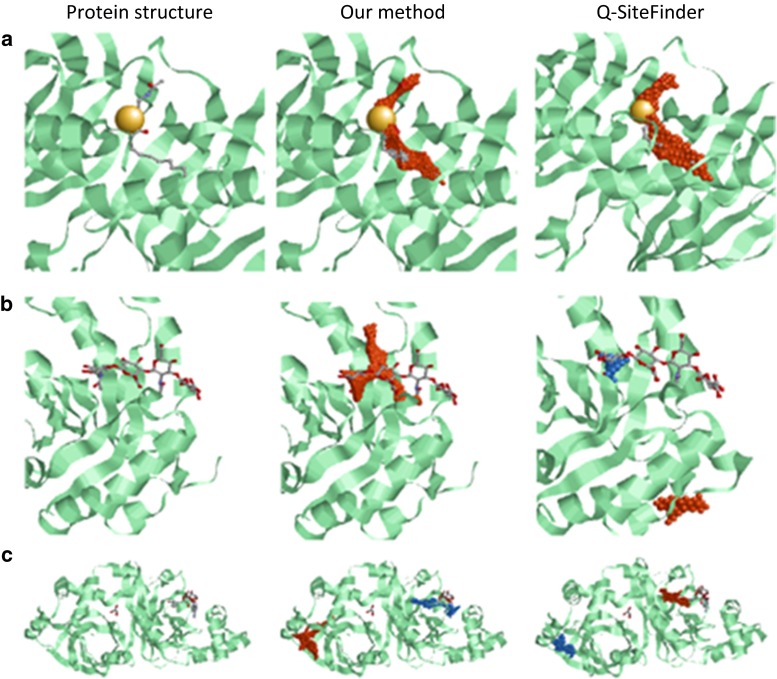
Comparison of prediction results from our proposed method and Q-SiteFinder (for ligand-binding space prediction). From *left*, crystal structures of protein–ligand complex, prediction results from the proposed method, and prediction results from Q-SiteFinder. *Green* proteins; *gray* ligands; *orange* clusters in the first prediction rank; and *blue* clusters in the lower than second prediction ranks. **a** Prediction results of PDBID: 1mka (ligand: 2-decenal *n*-acetyl cysteine). Precision of the proposed method: 93.2 %, precision of Q-SiteFinder: 86.3 %. Ligand-binding site was predicted with higher precision by the proposed method. **b** Prediction results of PDBID: 1fcv (*n*-acetyl-*D*-glucosamine). Precision of the proposed method: 59.0 %, precision of Q-SiteFinder (blue): 100 %. The cluster correctly predicted by Q-SiteFinder was in the sixth prediction rank, whereas that by our proposed method was in the first prediction rank. **c** Prediction results of PDBID: 2wn7 (ligand: nicotinamide adenine dinucleotide). Precision of the proposed method (*blue*): 38.3 % and precision of Q-SiteFinder: 34.7 %. The cluster correctly predicted by our proposed method was in the second prediction rank

**Fig. 3:**
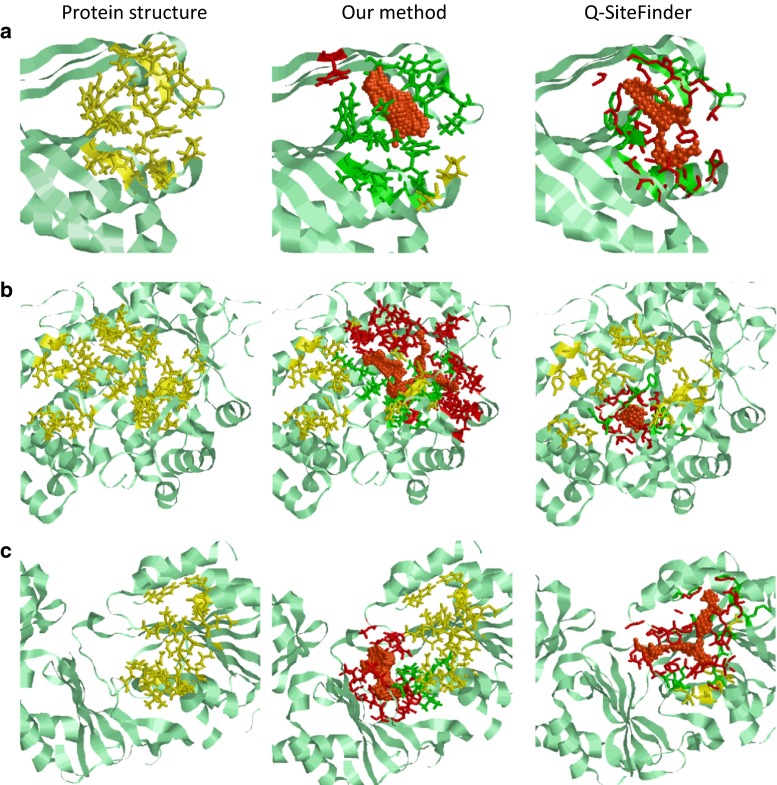
Comparison of prediction results from our proposed method and Q-SiteFinder (for ligand-binding residue prediction). From *left*, crystal structures of protein–ligand complex, prediction results from the proposed method, and prediction results from Q-SiteFinder. *Orange* cluster predicted in the first prediction rank, *green* correctly predicted residues (true positive, TP), *red* incorrectly predicted residues (false positive, FP), and *yellow* unpredicted residues (false negative, FP). **a** Prediction results of PDBID: 1y2q. Proposed method: *S*_residue_, 93.3 % and MCC, 0.701. Q-SiteFinder: *S*_residue_, 86.3 % and MCC, 0.859. The results show that many residues were not predicted by the proposed method, whereas Q-SiteFinder incorrectly predicted many residues. **b** Prediction results of PDBID: 1cwy. Proposed method: *S*_residue_, 68.2 % and MCC, 0.667. Q-SiteFinder: *S*_residue_, 10.0 % and MCC, 0.078. Q-SiteFinder formed a cluster deviating to the *left* of the correct binding site. **c** Prediction results of PDBID: 2b78. Proposed method: *S*_residue_, 15.4 % and MCC, 0.096. Q-SiteFinder: *S*_residue_, 61.3 % and MCC, 0.662. Our proposed method formed a cluster deviating to the left of the correct binding site. MCC, Matthew’s correlation coefficient

Figures 2 and 3 show examples of prediction results from both our proposed method and Q-SiteFinder.

## Discussion

The main differences between the proposed method and Q-SiteFinder include force field parameters, energy threshold of clustering, and the use of amino acid conservation.

With respect to the force field parameter, for ligand-binding site prediction based on interaction energy calculation, we found that prediction with AMBER parm94 yields a higher success rate than GRUB [17], employed by Q-SiteFinder [18].

The energy threshold of clustering is an important parameter governing the size of a cluster. If it is too low, clusters are unlikely to be formed and sensitivity will be degraded; conversely, if it is too high, more probes with small interaction energies can be formed. Q-SiteFinder uses 1.4 kcal/mol as the fixed energy threshold. However, the interaction energy between a protein and a probe should be different for each protein. Therefore, we instead used the upper limit of the number of probes in a cluster, *P*_num_, to restrict the size of the cluster. Of course, this solution is not perfect: for proteins that bind small ligands, our method tends to generate clusters larger than the size of the ligands. This tendency affects the precision. As seen in Tables 1 and 2, the average precision and average *S*_residue_ of our method without the amino acid conservation score (*C*_score_) were lower than those of Q-SiteFinder. This is ascribed to the use of the fixed *P*_num_ value larger than the size of the small ligands.

**Fig. 4:**
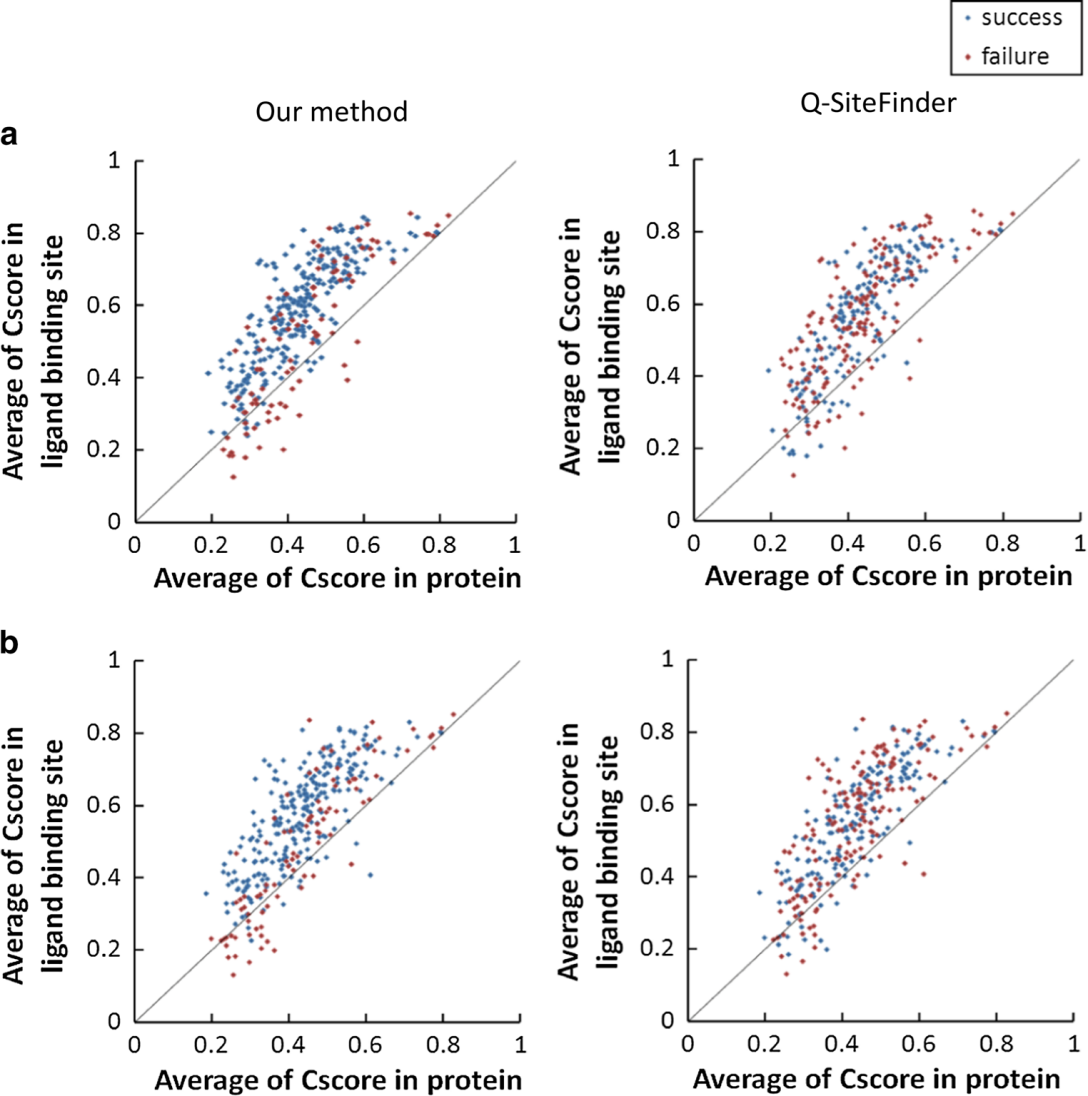
Correlation of the mean values of amino acid conservation (*C*_score_) around ligand-binding sites with the mean value of amino acid conservation in entire protein sequences. *Blue* *points* represent successfully predicted proteins, whereas *red points* represent proteins for which prediction failed. *Lines* correspond to equal mean values of amino acid conservation between the entire sequence and around ligand-binding sites. **a** Prediction results for 342 ligand-bound structures (for ligand-binding space prediction). **b** Prediction results for 348 ligand-unbound structures (for ligand-binding residue prediction)

In Fig. 4, the mean values of amino acid conservation (*C*_score_) around ligand-binding sites are plotted against the mean value of amino acid conservation in entire protein sequences. In proteins with multiple ligand-binding sites, residues around all binding sites were treated as ligand-binding residues. Points above the lines indicate proteins for which the mean values of amino acid conservation around ligand-binding sites are greater than those of the entire sequence. In contrast, points below lines indicate proteins for which mean values of amino acid conservation around ligand-binding sites are smaller than those of the complete sequence. This figure shows that in both ligand-bound proteins (Fig. 4a) and ligand-unbound proteins (Fig. 4b), there are more proteins lying above than below the lines, irrespective of the feasibility of prediction, that is, most of the proteins used for evaluation in the present study showed higher amino acid conservation around ligand-binding sites than in entire sequences. The numbers of such proteins were 309 (=244 + 65) for ligand-bound structures and 315 (=247 + 68) for ligand-unbound structures.

Comparison of the prediction results of our proposed method and Q-SiteFinder indicates that several red points lying above the lines in Q-SiteFinder graphs have changed to blue points in the graphs produced by our proposed method. In particular, when the difference between amino acid conservation between entire sequences and ligand-binding sites is high (proteins located further from the line in the upper left graph), more proteins were successfully predicted in the present study. These results show that our proposed prediction method is likely to succeed for proteins in which the amino acids around the ligand-binding sites are highly conserved.

However, these results do not indicate that higher amino acid conservation around the ligand-binding site will always result in higher prediction precision. The optimal weights, which determine the contribution of the van der Waals energy term (*E*^van^) and the amino acid conservation term (*C*_score_), depend on the training dataset but the results show that the contribution of van der Waals energy is larger than the amino acid conservation; the amino acid conservation alone does not yield a better result than the van der Waals energy alone.

### Proteins for which prediction failed

**Fig. 5:**
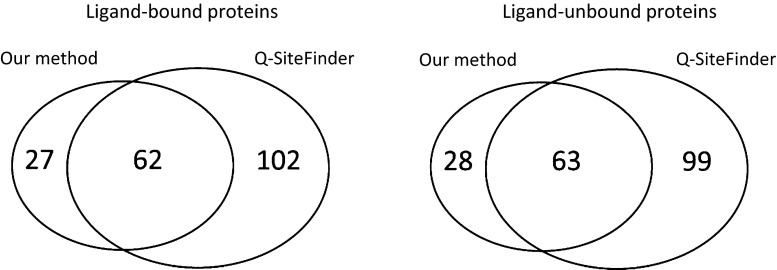
Numbers of proteins for which prediction by Q-SiteFinder and our proposed method failed. (*Left*) Prediction results for 342 ligand-bound structures (for ligand-binding space prediction). (*Right*) Prediction results for 348 ligand-unbound structures (for ligand-binding residue prediction). For both methods, the numbers of failed proteins are similar for ligand-bound and ligand-unbound proteins

Figure 5 indicates the number of proteins for which prediction by our proposed method, Q-SiteFinder, or both methods failed. Both the proposed method and Q-SiteFinder failed to predict 62 ligand-bound and 63 ligand-unbound structures. In these proteins, the prediction of ligand-binding site based on interaction energy calculation was indicated to be difficult. In addition, prediction of almost 30 proteins of either type failed only when the proposed method was used.

Prediction failure can be attributed to two major causes. First, prediction failed when there was no ligand at the predicted site, but one of its homologue proteins has a ligand at the corresponding site (i.e., the predicted site was one of the potential ligand-binding sites). Among failed structures, 19 ligand-bound structures and 23 ligand-unbound structures were categorized as this type. Accordingly, 5–7 % of proteins in the dataset for which prediction of our method failed were practically successful. Second, the conservation of ligand-binding sites was low, and another site with higher conservation was incorrectly predicted. Among failed structures, 11 proteins were categorized as this type.

## Summary

Our method predicts ligand-binding sites using van der Waals energy and amino acid conservation calculated from alignment with homologous sequences. In a comparison of our proposed method with Q-SiteFinder, a ligand-binding site prediction method based on interaction energy calculation, a much higher success rate (proportion of successfully predicted proteins) was obtained by our method than that by Q-SiteFinder. The present study indicates that amino acid conservation is an important factor in the success of ligand-binding site prediction, and that its combination with interaction energy calculation enables more precise site prediction. One of the binding sites of proteins with multiple ligand-binding sites was correctly predicted; however, applying the proposed method to a protein with less-conserved ligand-binding sites sometimes resulted in failure. This result suggests that in some cases, when the conservation of ligand-binding sites is low, the weighting of amino acid conservation can result in prediction failure. In this study, we did not explicitly filter out the suspicious ligands. The selection of ligands using the results of B-factor and LLDF described in Supplementary Material would be a future work.

## Electronic supplementary material

Below is the link to the electronic supplementary material.

Supplementary material 1 (DOCX 53 kb)

## Abbreviations

PDB: Protein data bank
PSSM: Position-specific scoring matrix
SBDD: Structure-based drug design
3D: Three-dimensional

## References

[1] Kuntz ID, Blaney JM, Oatley SJ, Langridge R, Ferrin TE (1982). A geometric approach to macromolecule-ligand interactions. J Mol Biol.

[2] Levitt DG, Banaszak LJ (1992). POCKET: a computer graphics method for identifying and displaying protein cavities and their surrounding amino acids. J Mol Graph.

[3] Hendlich M, Rippmann F, Barnickel G (1997). LIGSITE: automatic and efficient detection of potential small molecule-binding sites in proteins. J Mol Graph Model.

[4] Huang B, Schroeder M (2006). LIGSITEcsc: predicting ligand binding sites using the Connolly surface and degree of conservation. BMC Struct Biol.

[5] Goodford PJ (1985). A computational procedure for determining energetically favorable binding sites on biologically important macromolecules. J Med Chem.

[6] Laurie ATR, Jackson RM (2005). Q-SiteFinder: an energy-based method for the prediction of protein-ligand binding sites. Bioinformatics.

[7] Kulharia M, Bridgett SJ, Goody RS, Jackson RM (2009). InCa-SiteFinder: a method for structure-based prediction of inositol and carbohydrate binding sites on proteins. J Mol Graph Model.

[8] Magliery TJ, Regan L (2005). Sequence variation in ligand binding sites in proteins. BMC Bioinform.

[9] Capra JA, Laskowski RA, Thornton JM, Singh M, Funkhouser TA (2009). Predicting protein ligand binding sites by combining evolutionary sequence conservation and 3D structure. PLoS Comput Biol.

[10] Dessailly BH, Lensink MF, Orengo CA, Wodak SJ (2008). LigASite–a database of biologically relevant binding sites in proteins with known apo-structures. Nucleic Acids Res.

[11] Krissinel E, Henrick K (2007). Inference of macromolecular assemblies from crystalline state. J Mol Biol.

[12] Canutescu AA, Shelenkov AA, Dunbrack RL (2003). A graph-theory algorithm for rapid protein side-chain prediction. Protein Sci.

[13] Cornell WD, Cieplak P, Bayly CI, Gould IR, Merz KM, Ferguson DM, Spellmeyer DC, Fox T, Caldwell JW, Kollman PA (1995). A second generation force field for the simulation of proteins, nucleic acids, and organic molecules. J Am Chem Soc.

[14] Wang K, Samudrala R (2006). Incorporating background frequency improves entropy-based residue conservation measures. BMC Bioinform.

[15] Capra J, Singh M (2007). Predicting functionally important residues from sequence conservation. Bioinformatics.

[16] Schmidt T, Haas J, Gallo Cassarino T, Schwede T (2011). Assessment of ligand-binding residue predictions in CASP9. Proteins.

[17] GRID (2016) http://www.moldiscovery.com/software/grid/. Accessed 21 Jan 2016

[18] Morita M, Nakamura S, Shimizu K (2008). Highly accurate method for ligand-binding site prediction in unbound state (apo) protein structures. Proteins.

