# Supplementary material for: Development of a protein–ligand-binding site prediction method based on interaction energy and sequence conservation
Source: J Struct Funct Genomics. 2016 Jul 11;17(2):39–49. doi: 10.1007/s10969-016-9204-2 (PMC5002282; doi:10.1007/s10969-016-9204-2)
Supplement: Supplementary file 1 — Supplementary material 1 (DOCX 53 kb) [file 10969_2016_9204_MOESM1_ESM.docx]

Online Resource 1

Development of a protein-ligand binding site prediction method based on interaction energy and sequence conservation

Journal of Structural and Functional Genomics

Hiroto Tsujikawa, Kenta Sato, Kazuya Sumikoshi, Shugo Nakamura, Tohru Terada, and Kentaro Shimizu

Corresponding author:

Kentaro Shimizu

Professor

E-mail: shimizu@bi.a.u-tokyo.ac.jp

We first checked for ligands with partial occupancy and found only 195 ligands with one or more atom with occupancy less than 1.0. The *B*-factor was then calculated for these ligands using protein atoms within 10 Å of the ligand (using ligand atoms to find all protein atoms that are within this distance). We found that 144 of the 195 ligands (78%) have average *B*-factor of 30 or less. Fifty-one ligand structures, among 1,332 ligands in our list, could require attention using this method. Table S1 includes a list of PDBIDs of 382 ligand-bound and ligand-unbound structures used in this study. The proteins with the above 51 ligands as bound structures are marked.

We also performed analysis of Local Ligand Density Fit value (LLDF). The ligand structures from PDB were first verified using PDB’s structure analysis report (for LLDF). The structures were then checked for partial occupancy and the B-factor value of surrounding protein atoms. Using the PDB validation reports, we found that 1,245 of the ligands, among 1,332 ligands in our list, have LLDF values, whereas 68% (847 of 1,245) of their LLDF values are less than or equal to 2. Although some values are large, the mean LLDF value of all LLDF values was 2.034. The results are shown in Figure S1.


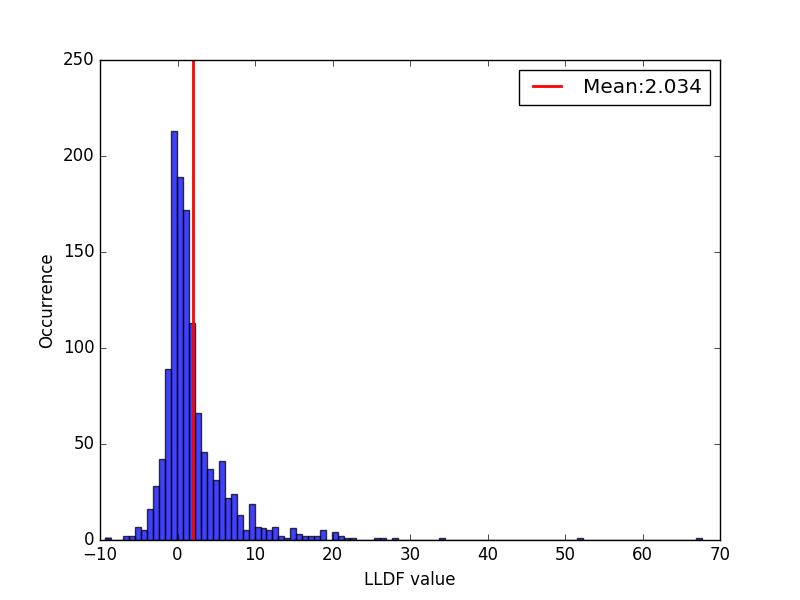


Figure S1 Histogram of Local Ligand Density Fit (LLDF) values in our dataset.

**Table S1** PDBIDs of 382 ligand-bound and ligand-unbound structures used in the present study.

| Ligand-  bound | Ligand-  unbound |  | Ligand-  bound | Ligand-  unbound |  | Ligand-  bound | Ligand-  unbound |  | Ligand-  bound | Ligand-  unbound |
| --- | --- | --- | --- | --- | --- | --- | --- | --- | --- | --- |
| 148l | 180l |  | 1jcl | 1p1x |  | 1rid | 1g40 |  | 1y3i | 1u0t |
| 1a26 | 2paw |  | 1jif | 1qto |  | 1rj4 | 1rj1 |  | 1ybu | 1ybt |
| 1arc | 1arb |  | 1jq3 | 1inl |  | 1rn8 | 1dup |  | 1yfr | 1riq |
| 1b16 | 1a4u |  | 1jxm | 1jxo |  | 1rsd | 1dhn |  | 1ymg | 2b6p |
| 1b8v | 1b8p |  | 1jyl | 1jyk |  | 1rya | 2gt2 |  | 1ytj | 1az5 |
| 1blc | 3blm |  | 1k0n | 1k0m |  | 1rzu | 1rzv |  | 1ytm | 1yvy |
| 1bq4 | 4pgm |  | 1k54 | 1ewz |  | 1s3f | 1s2l |  | 1yvp | 1yvr |
| 1bsu | 1az3 |  | 1kaq | 1kam |  | 1s7l | 1s7k |  | 1zdf | 1zcu |
| 1c1h | 1ak1 |  | 1kgz* | 1khd |  | 1sgc | 2sga |  | 1znx | 1znw |
| 1c5i | 1c5h |  | 1kpf | 1kpa |  | 1sqf | 1sqg |  | 1zt9 | 1p6z |
| 1cen | 1ceo |  | 1kq4 | 1o24 |  | 1sth | 1ey0 |  | 1zu0 | 1zty |
| 1cg0 | 1ade |  | 1kwc | 1kwb |  | 1su2 | 1sjy |  | 2a4z | 1e8y |
| 1cqf | 1c48 |  | 1l6l | 2ou1 |  | 1svi | 1sul |  | 2a70 | 2a6z |
| 1dai | 1byi |  | 1lby | 1lbv |  | 1swk | 1swh |  | 2ahw | 2ahu |
| 1dcp | 1dco |  | 1llo | 2hvm |  | 1szj* | 1crw |  | 2aib | 2a8f |
| 1e3v | 1opy |  | 1lsp | 1gbs |  | 1the | 1cpj |  | 2art | 2c7i |
| 1e4g | 1e4f |  | 1ltz | 1ltu |  | 1tjw | 1tjv |  | 2ate | 1qcz |
| 1e5q | 1e5l |  | 1lxm | 1f1s |  | 1tkg | 1tje |  | 2b3d | 2amj |
| 1ein | 1tib |  | 1mbz | 1m1z |  | 1tox | 1sgk |  | 2b99* | 2b98 |
| 1epb | 1epa |  | 1mka | 1mkb |  | 1tvp | 1tvn |  | 2baw | 2gwx |
| 1esw | 1cwy |  | 1mrl | 1mr7 |  | 1txc | 1tw0 |  | 2bl1 | 2j8n |
| 1eyr* | 1ezi |  | 1mu0 | 1mtz |  | 1tyx | 1tyv |  | 2bof | 2boe |
| 1f12 | 1f14 |  | 1mxi | 1j85 |  | 1tzx | 1tzv |  | 2c4h | 1qid |
| 1f74 | 1f5z |  | 1n08 | 1n05 |  | 1u2g | 1l7d |  | 2c6z | 2ci3 |
| 1fcv | 1fcq |  | 1nco | 1noa |  | 1u80 | 1u7u |  | 2c96 | 2bjw |
| 1ffq | 1edq |  | 1ndi | 1ndb |  | 1ual | 1uaj |  | 2cht | 2chs |
| 1fk4 | 1mzl |  | 1no6 | 1oem |  | 1ucd | 1bk7 |  | 2cwf | 1wtj |
| 1foa | 1fo9 |  | 1nxd | 1dq0 |  | 1uf8 | 1uf4 |  | 2czv | 1v77 |
| 1fth | 1ftf |  | 1nzc | 1nxm |  | 1um0 | 1umf |  | 2d29 | 1ws9 |
| 1fxs | 1gfs |  | 1ofa | 1ofp |  | 1uy0 | 1uxz |  | 2d5a | 2d59 |
| 1g6c | 1g4e |  | 1og1* | 1gy0 |  | 1v0y | 1v0s |  | 2dg3 | 2ppn |
| 1g7v | 1x8f |  | 1ogk | 1ogl |  | 1v3s | 1vfj |  | 2dkc* | 2dka |
| 1g97 | 1g95 |  | 1ogo | 1ogm |  | 1vh3* | 1vic |  | 2dpt | 2dps |
| 1ga0 | 1gce |  | 1ojz | 1ojq |  | 1vps | 1vpn |  | 2dtt | 2dj6 |
| 1goq | 1k6a |  | 1oxm | 1cex |  | 1wnz | 1wny |  | 2dve | 2e10 |
| 1goy | 1gou |  | 1oxv* | 1oxt |  | 1woo | 1wos |  | 2dxd | 2cwk |
| 1gsa | 1gsh |  | 1p4n | 1xix |  | 1wxg | 1wxf |  | 2dzb | 2dqw |
| 1guh | 1k3o |  | 1p5r | 1p5h |  | 1x2b | 1qtr |  | 2e0n | 2e0k |
| 1gwm | 1gwk |  | 1p77 | 1p74 |  | 1x55 | 1x56 |  | 2e1t | 2e1v |
| 1h74 | 1fwl |  | 1pfk* | 2pfk |  | 1x7p | 1x7o |  | 2e2r | 2zbs |
| 1hmp | 1z7g |  | 1pkk | 1ogh |  | 1xdh | 1lf4 |  | 2e3n | 2e3s |
| 1hnj | 1hnk |  | 1pnf | 1png |  | 1xny | 1xo6 |  | 2e4g | 2oam |
| 1i1h | 1f2v |  | 1pwd | 3pte |  | 1xpy | 1r0m |  | 2e5m | 2e0c |
| 1i7l | 1i7n |  | 1q51* | 1q52 |  | 1xqp* | 1xqo |  | 2e9z* | 1u09 |
| 1ig1 | 1jks |  | 1q8j | 1q7m |  | 1xtb | 1hm5 |  | 2ecu | 2ecr |
| 1ixo | 1ho1 |  | 1qji | 1iad |  | 1xvt | 1xk7 |  | 2f10 | 1snt |
| 1j39 | 2bgt |  | 1rb0 | 1hka |  | 1xwq | 1xw2 |  | 2f7i | 1f41 |
| 1j8r | 1j8s |  | 1rd9 | 1fgb |  | 1xz8 | 1non |  | 2f9w | 2f9t |
| 1j9l | 1ilv |  | 1rez | 2nwd |  | 1y2x | 1y2t |  | 2fa0* | 2f82 |
| 1jcg | 1jcf |  | 1ri4 | 1ri5 |  | 1y30 | 1w9a |  | 2fhk | 1ftr |
| 2fpc | 2fp8 |  | 2uyq | 2uyo |  | 3baz | 3ba1 |  | 3hit | 3his |
| 2fsi | 2fsf |  | 2v8l | 2vq4 |  | 3bts | 3btv |  | 3hiy | 3hj4 |
| 2fv1 | 2ahf |  | 2var | 2v78 |  | 3byn | 3byl |  | 3hp8 | 3hnx |
| 2fzs | 1tyf |  | 2vfc | 2vfb |  | 3c2f | 3c2e |  | 3hpi | 3kjt |
| 2g97 | 2g95 |  | 2vfl | 2vfy |  | 3cag | 3bue |  | 3hpq | 4ake |
| 2gg6 | 2gg4 |  | 2vu9 | 2vua |  | 3cb5 | 3cb6 |  | 3hvl | 3ctb |
| 2gte | 1ooi |  | 2w1a | 2qbv |  | 3cgy | 3cgz |  | 3hvo | 3gsz |
| 2gwh | 2ad1 |  | 2wea | 3app |  | 3cnm | 3ex9 |  | 3i0d | 3i0c |
| 2gz3 | 2gyy |  | 2wi7 | 1uyl |  | 3cq3 | 3cq1 |  | 3i6c | 3ik8 |
| 2hbl | 2hbj |  | 2wn7 | 2wn4 |  | 3crr | 3crm |  | 3i92 | 3i8s |
| 2hix | 2hiv |  | 2wu1* | 1fsf |  | 3ct0 | 3csr |  | 3i9j | 1r12 |
| 2hk1 | 2hk0 |  | 2wva | 2wvh |  | 3cu1 | 3caf |  | 3ido | 3ily |
| 2hl0 | 1y2q |  | 2wzm | 2wzt |  | 3cwk | 3d95 |  | 3ier | 3iep |
| 2hzq | 2hzr |  | 2x4r* | 2yxf |  | 3cxi* | 3i3i |  | 3iiq* | 1kn9 |
| 2i4n | 2i4l |  | 2x60 | 2x5s |  | 3d1g | 1mmi |  | 3in1 | 3h49 |
| 2i56 | 3ity |  | 2xew | 1ubi |  | 3d4p | 3d0o |  | 3ivm | 3iuj |
| 2iea | 2g67 |  | 2xoi | 1kf5 |  | 3dsr* | 2c61 |  | 3jyn | 3jyl |
| 2ihk | 2ex0 |  | 2xsq | 3cou |  | 3du4 | 3drd |  | 3kak | 3kaj |
| 2io9 | 2iob |  | 2y77 | 2dhq |  | 3duw | 3dul |  | 3kbn | 2gub |
| 2irx | 2iru |  | 2ylc | 1hk9 |  | 3dws | 1vju |  | 3kdi | 3kdh |
| 2j4e | 2car |  | 2yw2 | 2yya |  | 3e3s | 1thv |  | 3kjg | 3kje |
| 2j72 | 2j71 |  | 2yw9 | 1ulu |  | 3efv | 3etf |  | 3kp4 | 3kp7 |
| 2j8y | 2j7v |  | 2ywc | 2ywb |  | 3ej2 | 3eiz |  | 3ku1 | 3kr9 |
| 2jbs | 2jbr |  | 2yyu | 2yyt |  | 3ek5 | 3ek6 |  | 3kv8 | 3kx7 |
| 2jgv | 2q5r |  | 2z09 | 1wjg |  | 3evl | 3evh |  | 3lbz* | 1r29 |
| 2oec | 2qdk |  | 2z0k | 2cx5 |  | 3exs | 3exr |  | 3ldf | 2b78 |
| 2ovd | 2ove |  | 2z0y | 1v6z |  | 3f1k | 3f1l |  | 3ldk | 3lig |
| 2p4j | 1sgz |  | 2z1s | 2o9p |  | 3f47 | 2b0j |  | 3ll3 | 3gbt |
| 2pkk | 2pkf |  | 2za3 | 2zcg |  | 3fci | 1akz |  | 3lzz | 3loi |
| 2q6v | 2hy7 |  | 2zbq | 2nxc |  | 3ftf | 3ftd |  | 3m4e | 3m4d |
| 2q71 | 2q6z |  | 2zdq | 2yzg |  | 3fwr | 3fv6 |  | 3man | 1bqc |
| 2qeh | 2qev |  | 2zgm | 2zgl |  | 3g11 | 2gfv |  | 3maq | 3k5o |
| 2qo9 | 2gsf |  | 2zgz | 1mwk |  | 3g1x | 3g1s |  | 3muf | 1zuh |
| 2qtt | 2qsu |  | 2zhz | 2zhy |  | 3gd9 | 3gd0 |  | 3nk7* | 3nk6 |
| 2qv7 | 2qvl |  | 2zja | 2zj8 |  | 3gdl | 3gdk |  | 3nri* | 2zco |
| 2qx7 | 2qys |  | 2zu3 | 2zty |  | 3gh6 | 3f6f |  | 3nxv | 1pdb |
| 2qyq | 1bd9 |  | 2zu4 | 2h2z |  | 3gid | 3glk |  | 3ol2 | 1olz |
| 2r68 | 2r60 |  | 3a0t | 3a0y |  | 3gpl | 3e1s |  | 3p0x | 3e5b |
| 2r7a* | 2rg7 |  | 3a2s | 3a2r* |  | 3gpo | 3gpg |  | 3pfr | 3n6j |
| 2rfh | 1arl |  | 3a5r | 3a5q |  | 3gqk | 3gqh |  | 3pr8 | 3lxz |
| 2rjc | 2rjd |  | 3aaq | 3aap |  | 3gyh | 3gva |  | 3ts1 | 2ts1 |
| 2rk2 | 2gqv |  | 3adp | 3ado |  | 3h2k | 3h2g |  | 3tu9 | 1zah |
| 2rkm | 1rkm |  | 3b3f | 3b3g |  | 3h39 | 3h38 |  | 3ueu | 1b8e |
| 2rmb | 3k0m |  | 3b4y | 3c8n |  | 3h72 | 3h71 |  | 5gal | 1bkz |
| 2sim | 2sil |  | 3b6a | 2opt |  | 3ha5 | 2fk7 |  |  |  |
| 2tpi | 1tgn |  | 3b6r | 3dre |  | 3hbd | 3hbh |  |  |  |
